# Isolated Diastolic Hypertension Associated Risk Factors among Chinese in Anhui Province, China

**DOI:** 10.3390/ijerph120404395

**Published:** 2015-04-22

**Authors:** Yanchun Wang, Fengjun Xing, Rongjuan Liu, Li Liu, Yu Zhu, Yufeng Wen, Wenjie Sun, Ziwei Song

**Affiliations:** 1 School of Public Health, Wannan Medical College, Wuhu 241002, China; E-Mails: wangyanchun2006@163.com (Y.W.); xfj_8@live.com (F.X.); 18575582924@163.com (R.L.); 18255310119@wnmc.edu (L.L.); kutuomonk@foxmail.com (Y.Z.); wyf@wnmc.edu.cn (Y.W.); 2 The Department of Pathology, School of Medicine, The Fourth Affiliated Hospital, Zhejiang University, Yiwu 322000, China; 3 School of Food Science, Guangdong Pharmaceutical University, Zhongshan 528458, China; 4 School of Public Health and Tropical Medicine, Tulane University, New Orleans, LA 70112, USA; E-Mail: zsong1@tulane.edu

**Keywords:** isolated diastolic hypertension, linear mixed model, case-control study

## Abstract

Objective: To explore potential risk factors of isolated diastolic hypertension (IDH) among young and middle-aged Chinese. Methods: A community-based cross-sectional study was conducted among 338 subjects, aged 25 years and above, using random sampling technique. There were 68 cases of IDH, 46 cases of isolated systolic hypertension (ISH), 89 cases of systolic and diastolic hypertension (SDH), and 135 of subjects with normal blood pressure. Cases and controls were matched on sex by frequency matching. Demographic characteristics, blood pressure and other relevant information were collected.Results: Compared with controls, patients with IDH and ISH had significant higher level of triglyceride, high density lipoprotein, blood glucose and body mass index (BMI) (*p* < 0.05); while patients with SDH had significantly higher level of total cholesterol, triglyceride, glucose and BMI (*p* < 0.05). Linear mixed effects model showed that drinking tea, family history of hypertension (FHH), higher blood glucose, triglyceride and low density lipoprotein were related with elevated diastolic blood pressure (DBP) (*p* < 0.01); HFH, blood glucose, creatinine and BMI have positive effect on systolic blood pressure (SBP) (*p* < 0.05). Conclusions: Drinking tea, FHH, high levels of triglyceride, high density lipoprotein, blood glucose and BMI are associated with IDH among young and middle-aged Chinese.

## 1. Introduction

Isolated diastolic hypertension (IDH), as unrecognized subtype of hypertension, is defined as DBP more than 90 mmHg and systolic BP less than 140 mmHg [1]. Of note, IDH had a high prevalence among younger age groups, especially in developing countries [2]. Although IDH carried a low risk of cardiovascular mortality [3], it is associated with an increased cardiovascular risk [4]. For example, DBP and IDH drive coronary risk in younger subjects in China [5]. Moreover, IDH is more likely to develop to SDH or ISH [6] which increase cardiovascular mortality [7].

Previous study indicated that higher levels of BMI, glucose, and uric acid concentrations were potential risk factors for IDH [8]. However, there are inconsistent reports concerning the risk factors of IDH [9,10]. For example, Zhuo *et al*. found that unreasonable diet structure was the main risk factor for IDH in exploring the differences between ISH and IDH risk factors among 20,364 patients (>35 years) [11], while the Framingham study showed those people who smoked, drank, and did not exercise were more likely to suffer from IDH [12]. Franklin *et al.* studied 5968 of people aged above 18 and found that in untreated, while at the same time in patients with hypertension and metabolic syndrome, IDH patients with metabolic syndrome risk ratio (14.7) was higher than that of ISH (10.2) and SDH (12.2) (*p* < 0.01), the probability of the metabolic syndrome is nearly 15 times as large as the ideal blood pressure [6]. 

Symptoms of IDH are less likely to be addressed by doctors or patients in China [13]. Thus, most non-newly-diagnosed IDH patients had ISH and SDH [10]. As IDH, ISH, and SDH are not independent, there could be the internal relations. It is difficult to detect the potential risk factors for IDH. Likewise, linear mixed effect model is a new method to deal with the complicated actualize the results of the hypothesis test since considers data aggregation, using corresponding iterative methods to obtain effective estimation and provide correct standard error. In addition, most studies on IDH is hospital-based and recruited people from hospital patients which could be limited by selection bias [14]. 

Therefore, we conducted a community-based study and used linear mixed effect model to explore the risk factors of newly diagnosed IDH among young and middle-aged Chinese.

## 2. Methods

### 2.1. Study Population

Participants were recruited from March to June in 2014 among a hospital physical examination population among whom 66.3% came from the community of Ma’anshan City, Auhui Province, China. Two hundred and three cases (68 IDH, 46 ISH, 89 SDH ) were selected from all of the hypertension patients according to the inclusion criteria, and 135 controls with normal blood pressure matched on sex by frequency matching were sampled by simple random sampling in the same certain period of time. All subjects gave their informed consent for inclusion before they participated in the study. The study was conducted in accordance with the Declaration of Helsinki, and the protocol was approved by the Ethics Committee of Wanna Medical College (NO.: 2014023). 

### 2.2. Research Methods

(1) Questionnaire survey: A self-administered questionnaire was used to collect information on demographic characteristics, including medical history, family history of hypertension, health-related knowledge, dietary pattern, exercise, alcohol consumption, smoking, drinking tea status, socioeconomic status, etc. Trained investigators disseminated questionnaire to participants and provided instructions to them. 

(2) Physical examination: height, body weight and blood pressure were measured according the standard protocol. Blood pressure was recorded using an aneroid sphygmomanometer, the participants were in a quiet and warm room. Two readings taken at least 5 minutes apart were obtained for each participant, and the average blood pressure was calculated.

(3) Laboratory examination: 5 mL of fasting venous blood was used to measure fasting blood glucose (FBG), total cholesterol (TC), high density lipoprotein (HDL), low density lipoprotein (LDL), triglyceride (TG),uric acid (UA) and creatinine (Cr) (Hitachi 7600 automatic biochemical analyzer, Hitachi, Tokyo, Japan).

### 2.3. Sample Selection

(1) Hypertension group: according to the 2010 guideline of hypertension prevention and control: hypertension is defined as SBP ≥ 140 mmHg and/or DBP ≥ 90 mmHg without administrating any antihypertensive drugs [15]. (2) Subtypes are isolated diastolic hypertension (IDH): SBP < 140 mmHg, DBP ≥ 90 mmHg; isolated systolic hypertension (ISH): SBP ≥ 140 mmHg, DBP < 90 mmHg; systolic and diastolic hypertension (SDH): SBP ≥ 140 mmHg, DBP ≥ 90 mmHg; all participants in this group were aged between 25 and 60 and were all newly diagnosed patients with essential hypertension. Participants with secondary hypertension were excluded from the current study. (3) Control group: participants with SBP < 140 mmHg and DBP < 90 mmHg and without heart disease, brain disorders, kidney disease, liver disease, diabetes, cancer and other disease history were included in the control group. They all also aged from 25 to 60 and had normal ECG, urine routine, liver function, blood glucose.

### 2.4. Definition of Covariates

BMI category: body mass index (BMI) < 24.0 kg/m^2^ is defined as normal, 24.0–27.9 kg/m^2^ as overweight, greater than or equal to 28 kg/m^2^ as obesity [16]. Intensity of physical activities is defined as performing physical activities at least three times a week, at least 20 minutes each time. Smoking is defined as consuming more than one cigarette a day for more than one year. Alcohol drinking is defined as drinking alcohol one time per month, the amount was equivalent to drinking 50 mL and above each time. Participants with less than 6 hours sleep were identified as lack of sleep and those drinking tea at least three times per week was identified as tea drinkers.

### 2.5. StatisticalAnalysis

Data was entered into database established using Epi Data 3.02 with double entry. SPSS 13.0 was used for statistical analysis. Chi-square test and analysis of variance were used to test the differences of the demographic characteristic of subjects. The Student-Newman-Keuls analysis of variance was conducted to compare differences of serum lipid, renal function, blood glucose BMI, physical and behavioral factors among IDH, ISH, SDH and control group. In order to explore the impact factors of different types of hypertension, especially the IDH, we used multivariable analysis of linear mixed effect model; Statistical significance was defined as *p* < 0.05.

## 3. Results

### 3.1. General Information

A total of 68 IDH cases, 46 ISH cases, 89 SDH cases and 135 controls were included in the current study with male: female ratio of 2.29:1. The average age were 41.97 ± 7.87, 43.98 ± 9.09, 45.12 ± 7.45 and 41.14 ± 9.71 in the IDH, ISH, SDH and control group, respectively. The proportion of genders was comparable across the four groups. No significant difference was detected in marital status, household registration and educational background among IDH, ISH, SDH and control groups (*p* > 0.05), and details have been presented in Table 1.

### 3.2. Comparison of Serum Lipid, Renal Function, Blood Glucose and BMI 

We tested the distribution of serum lipid, renal function, blood glucose, and BMI among people with different types of hypertension. Table 2 showed that in the IDH group and ISH group, TG, HDL, FBG and BMI level were significantly higher compared with those in control group (*p* < 0.05 or *p* < 0.01), while TC, TG, FBG and BMI level in SDH group were significantly higher compared with control group (*p* < 0.05 or *p* < 0.01); there were no significant differences of LDL UA and Cr among four groups.

**Table 1:** The demographic characteristic of subjects

Characteristics		IDH (n = 68)	ISH (n = 46)	SDH (n = 89)	Control (n = 135)	Total (n = 338)	χ^2^/F	*p*-Value
Sex	1	47 (69.12)	32 (69.57)	62 (69.66)	94 (69.63)	235	0.007	1.000
	2	21 (30.88)	14 (30.43)	27 (30.34)	41 (30.37)	103		
Average age		41.97 ± 7.87	43.98 ± 9.09	45.12 ± 7.45	41.14 ± 9.71	42.67 ± 8.78	4.050	0.008
Marital status	1	5 (7.35)	2 (4.35)	2 (2.25)	7 (5.19)	16	8.340	0.447
	2	61 (89.71)	44 (95.65)	86 (96.63)	125 (92.59)	316		
	3	2 (2.94)	0 (0.00)	0 (0.00)	3 (2.22)	5		
	4	0 (0.00)	0 (0.00)	1 (1.12)	0 (0.00)	1		
Educational background	1	3 (4.41)	3 (6.52)	4 (4.49)	5 (3.70)	15	3.004	0.964
	2	17 (25.00)	14 (30.43)	29 (32.58)	38 (28.15)	98		
	3	23 (33.82)	15 (32.61)	32 (35.96)	45 (33.33)	115		
	4	25 (36.76)	14 (30.43)	24 (26.97)	47 (34.81)	110		
Household registration	1	11 (16.18)	8 (17.39)	10 (11.24)	22 (16.307)	51	3.518	0.742
	2	21 (30.88)	14 (30.43)	36 (40.45)	52 (38.52)	123		
	3	36 (52.84)	24 (52.17)	43 (48.31)	61 (45.19)	164		

Notes: Marital status (1 = unmarried, 2 = married, 3 = divorce, 4 = widowed); household registration (1 = rural, 2 = town, 3 = city); cultural degree (1 = primary, 2 = junior, 3 = high, 4 = college degree and above).

**Table 2:** Comparison of serum lipid, renal function, blood glucose and BMI of each group.

Variable	IDH (n = 68)	ISH (n = 46)	SDH (n = 89)	Control (n = 135)	F	*p*-value
TC (umol/L)	5.14 ± 1.15	5.06 ± 1.31	5.35 ± 1.15 **^a^**	4.95 ± 0.94	2.373	0.070
TG (mmol/L)	2.26 ± 2.05 **^a^**	2.09 ± 1.67 **^a^**	2.04 ± 1.63 **^a^**	1.36 ± 0.78	7.619	<0.001
HDL (mmol/L)	1.23 ± 0.27 **^a^**	1.24 ± 0.33 **^a^**	1.28 ± 0.26 **^b^**	1.38 ± 0.29	5.587	0.001
FBG (mol/L)	6.23 ± 2.05 **^a^**	6.35 ± 1.79 **^a^**	6.43 ± 2.58 **^a^**	5.08 ± 0.73	13.530	<0.001
LDL (mmol/L)	2.68 ± 0.82	2.62 ± 1.15	2.79 ± 0.88	2.65 ± 0.61	0.654	0.581
UA (umol/L)	343.27 ± 89.15	351.56 ± 106.41	346.26 ± 106.03	329.48 ± 104.33	0.806	0.491
Cr (umol/L)	72.85 ± 14.83	69.72 ± 14.79	72.57 ± 25.34	71.74 ± 17.95	0.289	0.833
BMI (kg/m^2^)	24.18 ± 3.18 **^b^**	24.33 ± 3.14 **^b^**	24.21 ± 2.80 **^a^**	23.10 ± 2.67	4.155	0.007

Notes: Compared with the control group, **^a^** *p* < 0.01, **^b^** *p* < 0.05.

### 3.3. Comparison of Physical and Behavioral Characteristics

Table 3 shows the comparison of physical and behavioral characteristics in four groups. Compared with the control group, the proportion of participants with obesity and tea drinker in IDH group were significantly higher (*p* < 0.05), while there were more participants with overweight in ISH group than in control group (*p* < 0.05). In addition, the percent of FHH, and tea drinkers were significantly higher in SDH group than in the control group (*p* < 0.05 or *p* < 0.01).

### 3.4. The Linear Mixed Effects Model Analysis of Factors Influencing Blood Pressure

In linear mixed effect model, the different types of hypertension were treated as dependent variable, and those statistically significant variables in Table 1, Table 2 and Table 3 were treated as independent. From results of stepwise selection of linear mixed effect model, in Table 4 and Table 5, drinking tea, FHHFBG, TG and LDL had positive effect on DBP (*p* < 0.05 or *p* < 0.01); FHH, FBG and BMI had positive effect on SBP (*p* < 0.05 or *p* < 0.01); and Cr had negative effect on SBP (*p* < 0.05) and it is the protective factor of SBP.

**Table 3:** Comparison of physical and behavioral of each group.

Variable	IDH (n = 68)	ISH (n = 46)	SDH (n = 89)	Control (n = 135)	χ^2^	*p*-Value
Overweight	11 (16.18)	9 (19.57) **^a^**	11 (12.36)	13 (9.63)	4.641	0.200
Obesity	7 (10.29) **^a^**	4 (8.70)	4 (4.49)	3 (2.22)	7.668	0.042
FHH	18 (26.47)	15 (32.61)	32 (35.96) **^b^**	26 (19.25)	7.711	0.052
Smoking	36 (52.94)	26 (56.52)	41 (46.07)	79 (58.52)	3.508	0.320
Drinking	20 (29.41)	14 (30.43)	37 (41.57)	41 (30.37)	3.903	0.272
Drinking tea	40 (58.82) **^a^**	22 (47.83)	49 (55.06) **^a^**	56 (41.48)	6.987	0.072
Lack of Sleep	13 (19.12)	9 (19.57)	14 (15.73)	20 (14.81)	0.964	0.810
Physical Exercise	41 (60.29)	23 (50.00)	47 (52.81)	77 (57.04)	1.472	0.689

Notes: Compared with the control group, **^a^** *p* < 0.05, **^b^** *p* < 0.01.

**Table 4:** Analysis of impact factors of different types of hypertension for fixed effects.

Effect	Type	Tea	HFH	Estimate	Se	t	*p*-value
Type	1			72.5459	5.4185	13.39	<0.001
Type	2			105.27	7.6622	13.74	<0.001
Type **×** tea	1	1		3.0083	1.1394	2.64	0.009
Type **×** tea	1	2		−	−	−	−
Type **×** tea	2	1		1.5879	1.6112	0.99	0.325
Type **×** tea	2	2		−	−	−	−
Type **×** FHH	1		1	3.3353	1.2364	2.70	0.007
Type **×** FHH	1		2	−	−	−	−
Type **×** FHH	2		1	5.7527	1.7484	3.29	0.001
Type **×** FHH	2		2	−	−	−	−
TG **×** type	1			0.8861	0.4034	2.20	0.029
TG **×** type	2			0.4980	0.5704	0.87	0.383
FBG **×** type	1			0.8742	0.3145	2.78	0.006
FBG **×** type	2			2.1917	0.4447	4.93	<0.001
LDL **×** type	1			1.4471	0.6868	2.11	0.036
LDL **×** type	2			0.4233	0.9712	0.44	0.663
UA **×** type	1			−0.0021	0.0068	−0.30	0.761
UA **×** type	2			0.0171	0.0096	1.77	0.077
Cr**×** type	1			−0.0083	0.0340	−0.24	0.808
Cr **×** type	2			−0.0952	0.0481	−1.98	0.049
BMI **×** type	1			0.2692	0.2038	1.32	0.188
BMI **×** type	2			0.7140	0.2882	2.48	0.014

Notes: Type: type of blood pressure (1 = DBP, 2 = SBP); Tea: tea drinking (1 = drinking tea, 2 = never of occasional drinking tea); FHH family history of hypertension (1 = yes, 2 = no).

## 4. Discussion

In summary, drinking tea, HFH, blood glucose, triglyceride and low density lipoprotein were associated with elevated diastolic blood pressure (DBP); HFH, blood glucose, Cr and BMI had positive effect on systolic blood pressure (SBP). Meanwhile, different type of hypertension had different risk factors, of particular interest, drinking tea was a risk factor for IDH.

The target population of this study is young and middle-aged adults with an average age of 45 years old. Biologically, large artery elasticity functions well and only peripheral vascular resistance can increase diastolic blood pressure for population of this age [17].

**Table 5:** The fixed effects test of analysis of the impact factors of different types of hypertension.

Effect	F	*p*-Value
Type	110.07	<0.001
Type **×** tea	4.01	0.019
Type **×** FHH	5.63	0.004
Type **×** TG	2.72	0.068
Type **×** FBG	12.34	<0.001
Type **×** LDL	3.05	0.049
Type **×** UA	3.55	0.030
Type **×** Cr	2.99	0.052
Type **×** BMI	3.16	0.044

Notes: Type: type of blood pressure (1 = DBP, 2 = SBP); Tea: tea drinking (1 = drinking tea, 2 = never of occasional drinking tea); FHH family history of hypertension (1 = yes, 2 = no).

The high incidence of IDH among the population could be partly result from stress from people’s career and life, as well as, high prevalence of IDH risk factors including high TC, overweight, smoking, and drinking, *etc*. [18]. Of note, lack of physical activity among middle-aged Chinese is also associated with elevated blood pressure. Prevention programs on these factors are encouraged to promote healthy behaviors to control blood pressure and prevent hypertension.

The linear mixed effects model analysis indicated that tea drinking history, FHH, blood glucose, TC, LDL, UA and Cr are factors associated with blood pressure. Although the linear mixed effects model analysis of blood pressure was rarely reported in hypertension-related studies, family history, blood glucose, blood lipid have been frequently reported as impact factors for blood pressure [15]. The analysis also showed that tea drinking history, family history, blood glucose, TC and LDL had positive effects on diastolic blood pressure; family history, blood glucose, Cr and BMI have positive effects on systolic blood pressure, and the effect varies on systolic and diastolic blood pressure. Our study result is of reliability in that the mixed effects model takes into account the inherent relationship on different levels of the exposure, as well as intrinsic correlation among these variables [19]. Moreover, it is interesting finding that the history of tea drinking positively affects diastolic blood pressure, which is in line with findings of Frank’s study [20]. The effect of tea intake on blood pressure is controversial [21]. However, we did not collect information on the duration, quantity and types of tea drinking. Future research may further explore how duration, quantity and types of tea drinking may affect blood pressure.

Consistently, it is observed that compared with the control group, TC, HDL, blood glucose and BMI were statistically significantly higher among IDH group, ISH group and SDH group, which is consistent with other findings on IDH risk factors [2,22,23]. Therefore, more studies should be conducted to examine the mechanism of hypertension, especially among young and middle aged adults. Potential risk factors were reported as overweight, obesity, high blood lipids and other factors associated with excessive accumulation of adipose tissue in the outer peripheral vascular, which limits vasomotor of peripheral vascular and increases diastole of peripheral vascular resistance and directly lead to IDH [17]. Besides, elevated blood lipids may also increase the blood viscosity, resulting in increased peripheral resistance, thus elevating diastolic blood pressure [24]. Previous study showed that there were obvious differences in risk factors between IDH and ISH [22] while our results indicated there was no significant difference between IDH, SDH and ISH. This may be due to the small sample size. Our results were in line with previous studies in that a follow-up observational study that lasted for 10 years also reported that both IDH and ISH have similar clinical significance on predicting stroke [25]. It is necessary to start early intervention on IDH, since IDH might generally develop into SDH [9,26], which can increase the risk of CVD morbidity and mortality [27]. 

To our knowledge, our study is the first report on a positive relationship of tea drinking and IDH, although the effect size is small. However, our study has several limitations. For example, accurate information about the tea type, frequency, time, and quantity consumed are not available in present study. However, Anhui Province is the main production area of green tea, and the green tea is the most popular drink in the study setting. A survey in Tongcheng City (a neighboring city in Auhui Province) showed that more than 90% of the residents drink green tea [28]. Of note, in preliminary investigation of present study, only one participant was accustomed to red tea, so the consumption of different tea type was excluded in the later formal study. In addition, Chinese brew the tea with tea leaves (raw tea) in tea cups and then drink it during the whole day. Since the usage of tea leaves is certain, more cups are more likely to with the water consumption, not the tea’s effect (this is different from the Western habit of drinking tea made from tea bags). Future study with accurate data on the tea type, frequency, time, and quantity with IDH are warrant. 

## 5. Conclusions

Different type of hypertension had different risk factors, drinking tea, family history of hypertension, high levels of triglyceride, high density lipoprotein, blood glucose and BMI are risk factors of isolated diastolic hypertension among young and middle-aged Chinese.
